# Characterisation of an aerosol exposure system to evaluate the genotoxicity of whole mainstream cigarette smoke using the *in vitro* γH2AX assay by high content screening

**DOI:** 10.1186/2050-6511-15-41

**Published:** 2014-07-23

**Authors:** Carolina Garcia-Canton, Graham Errington, Arturo Anadon, Clive Meredith

**Affiliations:** 1 British American Tobacco, Group Research and Development, Regents Park Road, Southampton, Hampshire SO15 8TL, UK; 2 Department of Toxicology and Pharmacology, Universidad Complutense de Madrid, Madrid, Spain

**Keywords:** GammaH2AX, DNA damage, *In vitro*, Cigarette smoke, Genotoxicity, High content screening

## Abstract

**Background:**

The genotoxic effect of cigarette smoke is routinely measured by treating cells with cigarette Particulate Matter (PM) at different dose levels in submerged cell cultures. However, PM exposure cannot be considered as a complete exposure as it does not contain the gas phase component of the cigarette smoke. The *in vitro* γH2AX assay by High Content Screening (HCS) has been suggested as a complementary tool to the standard battery of genotoxicity assays as it detects DNA double strand breaks in a high-throughput fashion. The aim of this study was to further optimise the *in vitro* γH2AX assay by HCS to enable aerosol exposure of human bronchial epithelial BEAS-2B cells at the air-liquid interface (ALI).

**Methods:**

Whole mainstream cigarette smoke (WMCS) from two reference cigarettes (3R4F and M4A) were assessed for their genotoxic potential. During the study, a further characterisation of the Borgwaldt RM20S® aerosol exposure system to include single dilution assessment with a reference gas was also carried out.

**Results:**

The results of the optimisation showed that both reference cigarettes produced a positive genotoxic response at all dilutions tested. However, the correlation between dose and response was low for both 3R4F and M4A (Pearson coefficient, r = -0.53 and -0.44 respectively). During the additional characterisation of the exposure system, it was observed that several pre-programmed dilutions did not perform as expected.

**Conclusions:**

Overall, the *in vitro* γH2AX assay by HCS could be used to evaluate WMCS in cell cultures at the ALI. Additionally, the extended characterisation of the exposure system indicates that assessing the performance of the dilutions could improve the existing routine QC checks.

## Background

Cigarette smoke is a complex aerosol mixture consisting of more than 6,000 identified compounds that can be divided between the particulate phase, accounting for 4.5% of the total aerosol mixture mass, and the gas phase, accounting for 95.5% of the total aerosol mixture mass [1].

Testing and understanding the toxicity of cigarette smoke *in vitro* is a key step in the characterisation of modified tobacco products with potentially reduced harm. Adopting such strategies are in line with recommendations published by the Institute of Medicine “Clearing the Smoke” [2] and the World Health Organisation Framework convention on Tobacco Control (WHO FCTC) “The scientific basis of tobacco product regulation” [3].

Johnson and colleagues published a thorough review on the *in vitro* systems used to evaluate the toxicity of cigarette smoke [4]. In this review, the authors highlighted that the majority of tobacco-related *in vitro* toxicology studies are carried out in non-human cell models which are poorly validated for tobacco product comparison. They also concluded that better methods are needed, especially in relation to regulation and health claims. In the field of *in vitro* genotoxicity, the authors described that the evaluation of cigarette smoke has been carried out using mainly cigarette smoke condensate (CSC). However, CSC contains primarily particulate phase components compared to whole mainstream cigarette smoke (WMCS) which contains both particulate and gas phase components. We consider WMCS a more comprehensive exposure system to study toxicological effects *in vitro* (Table 1). Moreover, the *in vitro* genotoxicity data has been mainly obtained from animal-derived cell systems which are functionally very different from human-derived cells.

**Table 1:** **Physical forms of cigarette smoke used in *in vitro *** **testing**

**Name**	**Description**
Cigarette smoke condensate (CSC)	Comprises the particulate phase along with some vapour phase components. Generated by cold-trapping and condensation of smoke at extremely low temperatures. The condensed ‘tar’ is then typically extracted and diluted using acetone.
Cigarette smoke particulate matter (PM)	Comprises the particulate phase only. Particulates are typically collected by passing cigarette smoke through a Cambridge filter pad and are subsequently eluted from the filter pad using a solvent such as dimethylsulphoxide.
Cigarette smoke extract (CSE)	Comprises the particulate phase along with some vapour phase components. Generated by bubbling smoke through a liquid (e.g. phosphate-buffered saline or cell culture medium).
Whole mainstream cigarette smoke (WMCS)	Cells are directly exposed to smoke at the air-liquid interface. This is more representative of human exposure conditions, as cells are exposed to the gas and vapour phase components in an aerosol [5].

Table adapted from [6].

There are different *in vitro* genotoxicity assays that have been widely used in the assessment of tobacco products [4]. Some of the assays described such as the micronucleus or the mouse lymphoma assay focus on fixed DNA damage like chromosomal damage and mutations, their strengths and limitations have been previously summarised [7]. The comet assay is the only assay described by Johnson and colleagues that specifically detects DNA strand breaks. Although the assay is widely accepted and considered a mature method [8], it does not discriminate between single or double strand breaks and has shown high inter- and intra-experimental variation [9]. The *in vitro* γH2AX assay, on the other hand, is an emerging method for DNA damage detection. The phosphorylation of H2AX (named γH2AX) in response to DNA double strand breaks (DSB) was first described in 1998 [10] and has since been extensively investigated [11]. Some applications in which γH2AX has been used as a biomarker of DNA damage are pre-clinical drug development and clinical studies [12]. More recently, γH2AX has been suggested as a potential complement to the current battery of *in vitro* genotoxicity assays with applications in the assessment of cigarette smoke [7,13].

The aim of this study was to optimise the novel *in vitro* γH2AX assay by High Content Screening (HCS) that we had previously developed [14], in order to adapt it for the assessment of aerosols and to evaluate the genotoxic effect of two reference cigarettes in human lung-derived BEAS-2B cells at the air-liquid interface (ALI). The optimisation employs the Borgwaldt RM20S® smoking machine (RM20S®) as part of the exposure system that delivers WMCS to cells at the ALI [5].

The *in vitro* γH2AX assay has been previously used in the assessment of cigarette smoke using mainly CSC and indirect exposure to WMCS i.e. cell cultures that had a layer of media covering the cells continuously or intermittently during smoke exposure and therefore not considered true ALI exposure [15-19]. In general, flow cytometry has been the main method for γH2AX detection and analysis. In this study, we selected a microscopy-based automated scoring system known as HCS to acquire and quantify the γH2AX response after WMCS exposure to BEAS-2B cells at the ALI. WMCS was tested from two different cigarettes, 3R4F a reference cigarette from the University of Kentucky [20] and M4A a historical control used as internal reference in genotoxicity studies by British American Tobacco [21].

Overall, the results show that the *in vitro* γH2AX by HCS can be used as a high throughput tool to assess the genotoxic effect of WMCS in cultures exposed at the ALI. The results can be used to compare the genotoxic responses of different tobacco products. Furthermore, the optimised *in vitro* γH2AX assay for aerosol exposure could be a useful high content screening tool to assess the genotoxic potential of toxicants in gaseous form.

## Methods

### Cell culture

The human bronchial epithelial cell line BEAS-2B was purchased from ATCC (United States). Normal bronchial epithelium cells obtained from autopsy of non-cancerous individuals had been isolated, then infected with a replication-defective 12-SV40/adenovirus hybrid (Ad12SV40) and cloned to create an immortalised phenotype [22]. Cells were seeded into culture vessels that had been pre-coated with 0.03 mg/mL PureCol® bovine collagen solution (Nutacon, The Netherlands). Cells were then maintained in Bronchial Epithelial Growth Medium (BEGM®) at 37°C and 5% CO_2_ in a humidified incubator. BEGM® was prepared by supplementing Bronchial Epithelial Basal Medium with growth supplements provided in the manufacturer’s BEGM® SingleQuot® kit (Lonza Group Ltd., Belgium) containing: bovine pituitary extract, hydrocortisone, human epidermal growth factor, epinephrine, insulin, triiodothyronine, transferrin, gentamicin /amphotericin-B and retinoic acid. BEAS-2B cells were cultured and expanded in-house, the cells were used between passages 3 and 12 only. All cultures were negative for mycoplasma. Additionally, the cells were authenticated using the short tandem repeat profiling to confirm the nature of the cell cultures (LGC Standards, United Kingdom) [23].

### Smoking system

**Figure 1:**
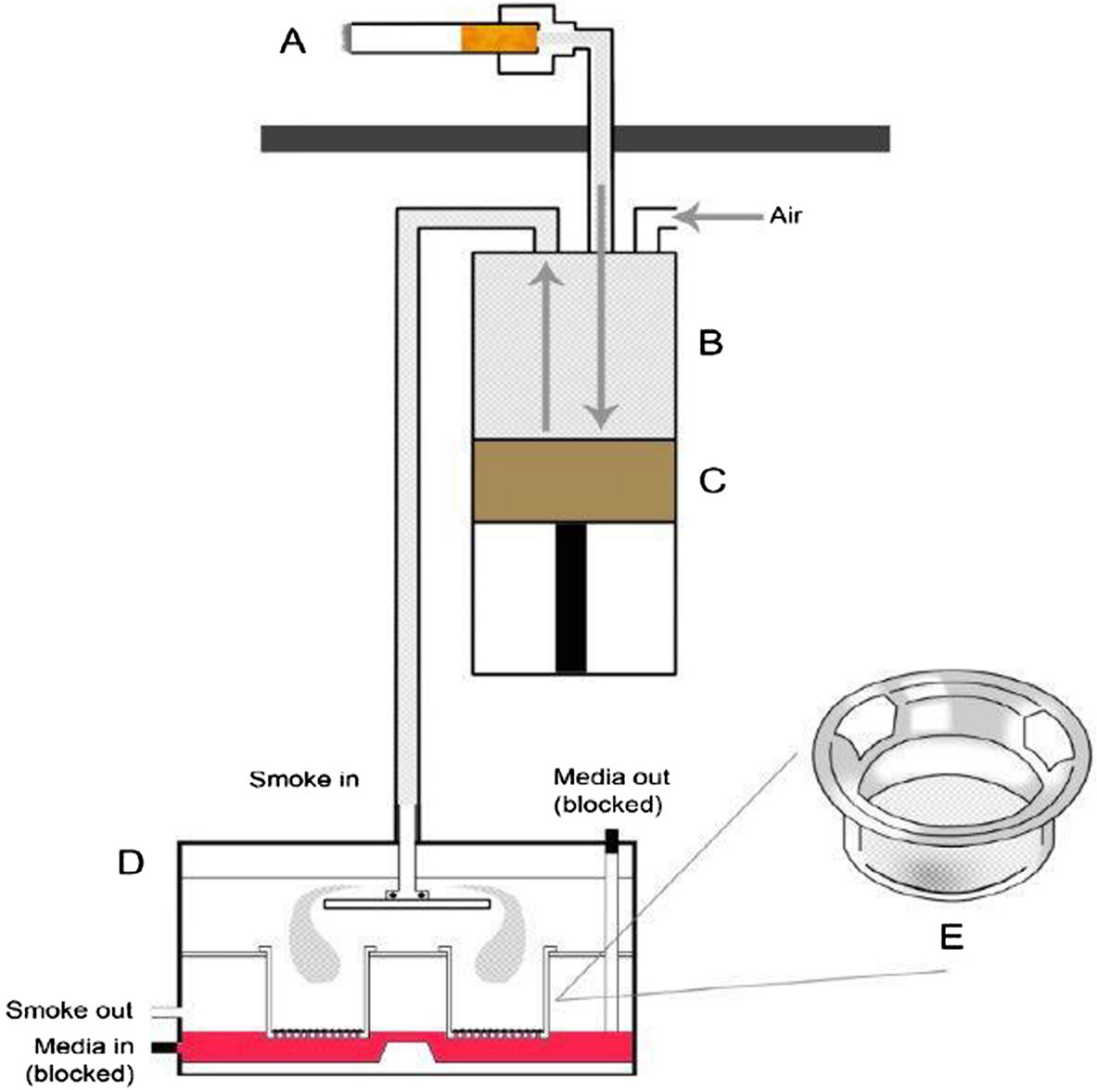
**Schematic representation of a single RM20S® syringe combined with British American Tobacco’s exposure chamber (UK patent publication WO 03/100417/ A1) (not to scale).** The RM20S® can smoke up to eight cigarettes simultaneously. **[A]** Cigarette holder with cigarette in place; **[B]** 150 mL glass syringe where cigarette smoke dilution in air is prepared; **[C]** Plunger; **[D]** Exposure chamber containing porous membrane inserts with cells seeded on top at the ALI **[E]** Transwell® insert representation. Figure adapted from [26].

The selection of the RM20S® 8-syringe smoking machine as the WMCS exposure system was based on previous *in vitro* studies [5,24] and thorough evaluations of precision, accuracy, repeatability and reproducibility [25,26]. The smoking exposure system is schematically represented in Figure 1. The RM20S® employs a dilution system that mixes WMCS with different proportions of air to generate a dilution ratio represented as 1 : X (smoke volume : air volume). Cigarettes are automatically loaded into cigarette holders (Figure 1A) where WMCS is drawn directly into the glass syringe and diluted with air taken from the laboratory environment (Figure 1B) following a multi-step process operated by a plunger (Figure 1C). The diluted WMCS is then delivered to an exposure chamber (Figure 1D) containing four Transwell® inserts with BEAS-2B cells seeded on top of the insert’s membrane (Figure 1E). At the time of the exposure the cells are directly exposed to WMCS at the air-liquid interface (ALI).

### Dilution performance evaluation

A range of dilutions were selected for this study from 1:25 to 1:20000 (smoke volume : air volume) (Table 2). The methodology employed by Kaur and colleagues used methane (CH_4_) as a reference gas standard with known parts per million (PPM) to compare syringe performance and has been adapted here to assess dilution performance [25]. For our experiments, three different methane reference standards in nitrogen were purchased from Air Products PLC (United Kingdom), 10% containing 100,000 PPM methane, 50% containing 500,000 PPM methane and 99.95% containing 1,000,000 PPM methane. The relevant reference gas was loaded into a sealed bag and connected directly to the smoking machine cigarette holder (Figure 1A). The dilution to be tested was then programmed into the RM20S® and then gas diluted following International Standard Organization (ISO) 3308:2012 puffing profile consisting of 35 mL puff volume, 2 sec puff duration, and 60 sec puff interval [27]. A second empty sealed bag was connected to the exhaust position in the place of the exposure chamber to collect the diluted gas (Figure 1D). Quantification of methane in PPM was performed with a 3010 MINIFID portable heated flame ionization detector total hydrocarbon analyser (Signal Group Ltd, United Kingdom) as per manufacturer’s instructions. Table 2 summarises details about dilutions, reference gas standard used per dilution and expected PPM. The laboratory environment was conditioned at 22 ± 2°C and 60 ± 5% Relative Humidity (RH).

**Table 2:** Range of dilutions, details of methane reference gas and expected PPM

**Dilutions**	**Expected PPM with 10% methane**	**Expected PPM with 50% methane**	**Expected PPM with 100% methane**
1:25	4000		
1:50	2000		
1:100	1000		
1:250	400		
1:500	200		
1:1000	100		
1:1500	67		
1:2000		250	
1:3000		167	
1:4000		125	
1:6000		83	
1:8000		63	
1:16000			63
1:20000			50

### Smoke exposure

Cigarettes were conditioned for a minimum of 48 hours prior to use (60 ± 3% relative humidity, 22 ± 1°C according to ISO 3402:1999) [28] and smoked continuously throughout the exposure on a RM20S® smoking machine (Borgwaldt KC, Germany) using a 35 ml puff volume drawn over 2 seconds, once every minute according to ISO 3308:2012 [27]. The smoking environment was conditioned at 22 ± 2°C and 60 ± 5% RH.

In this study two reference cigarettes were used to test whether the *in vitro* γH2AX assay by HCS could discriminate between products. The reference cigarette 3R4F supplied by the University of Kentucky, is a “US style” blended cigarette that delivers 9.4 mg tar and 0.7 mg nicotine under ISO conditions for cigarette smoking (ISO 3308:2012) [27]. Internal reference cigarette M4A is a flue cured cigarette that delivers 10 mg of tar and 1.0 mg nicotine under ISO conditions for cigarette smoking (ISO 3308:2012) [27].

### Controls

Etoposide (1 mM final) was used as a positive control during the experimentation (Sigma-Aldrich, United Kingdom). Etoposide is a well-known DNA-damaging compound and has previously been used in the *in vitro* γH2AX assay by HCS as a reference compound and positive control respectively [14,29]. Two different negative controls were used in this study; air control and incubator control. The air control was generated by the smoking machine to evaluate the quality of the air used to dilute the WMCS and mimic the exposure conditions. The incubator control evaluated the incubation conditions used to generate the positive controls.

### WMCS treatment, γH2AX immunostaining and imaging analysis

The methodology used during this study to detect and quantify γH2AX by HCS was previously described [14] with variations for the ALI exposure. In this study, cells were seeded on top of the membrane of collagen pre-coated 24-Transwell® plate (Corning Incorporated Life Sciences, Unites States) at a concentration of 1.2 × 10^5^ cells/mL and 500 μL of BEGM® were added underneath to keep the cells hydrated. The plates were then incubated overnight at 37°C in an atmosphere of 5% CO_2_ in air. At the time of treatment, the culture media was removed from the Transwell® membrane so the cells could be exposed directly at the ALI. Then, four inserts were transferred to each exposure chamber containing 25 mL of Dulbecco’s Modified Eagle Medium supplemented with 1% L-Glutamine and 0.5% penicillin/streptomycin (10000 IU/mL – 10000uG/mL). The exposure chambers were then placed in an incubator at 37°C and connected with plastic tubing to the smoking machine as represented in Figure 1D (smoke in/smoke out connectors). The smoking machine pre-programmed with the appropriate dilutions was set for a 3 hour exposure. We selected a 3 hour exposure as it is the minimum recommended in the International Conference on Harmonisation of Technical Requirements for Registration of Pharmaceuticals for Human Use (ICH guidelines) [30]. Following exposure, the inserts were placed in clean pre-labelled 24-well plates where the cells were fixed with 4% paraformaldehyde (100 μL/insert) and incubated for 15 minutes at room temperature. The fixed samples were processed for γH2AX immunostaining following manufacturer’s recommendation (ThermoScientific, United States).

Image acquisition was performed using the Cellomics ArrayScan® VTI platform (ThermoScientific, USA). Image analysis used the Target Activation Bioapplication software v.6.6.1.4. The protocol was set to count a minimum of 500 cells per insert, giving a minimum of 2000 cells per concentration tested. Nuclear DNA staining (Hoechst dye) was used to identify viable cells nuclei. These nuclei were used as the target areas for the measurement of γH2AX specific fluorescence intensity represented as absolute intensity units. Viable cell counts from negative controls were defined as 100% cell viability. The viable cell counts in the WMCS and etoposide treated samples were then compared to those in the negative control, and the percentage cell viability was calculated and referred to as Relative Cell Counts (RCC).

### Data analysis and criteria

#### *Dilution performance evaluation*

1-sample t-test was used to compare the results obtained in PPM for each dilution with the expected PPM. A variability of ±10% over the expected PPM was included afterwards as accepted measurement variation [31]. Repeatability and Reproducibility statistics were computed for all data points according to ISO 5725–2:1994 [32]. Experiments were replicated 3 times, with 6 repeats per dilution per experiment. Data analysis and graphical representations were performed with Minitab software v.16.

#### *WMCS genotoxicity evaluation*

The evaluation criteria used in this study (Table 3) was first described by Smart *et al.* for the analysis of γH2AX by flow cytometry [13] and recently applied by Garcia-Canton *et al.* for the analysis of γH2AX by HCS [14]. Experiments were replicated at least three times, with 4 repeats per dilution per experiment and graphical representation was performed using GraphPad Prism software v.6.01.

**Table 3:** **Genotoxicity evaluation criteria for the *in vitro *** **γH2AX assay by HCS**

**γH2AX response**	**Classification**
> 1.5-fold γH2AX @ RCC > 25%	Genotoxic (+)
< 1.5-fold γH2AX @ RCC 100-0%	Non-genotoxic (-)
> 1.5-fold γH2AX @ RCC < 25%	“False” positive; Cytotoxic-driven genotoxicity (C)
1.5-fold γH2AX @ RCC ≥ 25%	Equivocal (±)

Table adapted from [13].

## Results

The Borgwaldt RM20S® smoking machine combined with British American Tobacco’s chamber were used as an exposure system during the optimisation of the novel *in vitro* γH2AX assay by HCS for the evaluation of aerosols.

The initial steps in this optimisation included extending the QC checks of the RM20S® to include 14 dilution performance evaluations (Table 2). From the 10 dilutions generating accurate deliveries, 6 smoke dilutions were selected for further experiments based on range finder experiments (data not shown). The tested smoke dilutions covered a wide range of WMCS dilutions to assess the genotoxicity effect of two reference cigarettes (3R4F and M4A).

### Dilution performance evaluation

**Figure 2:**
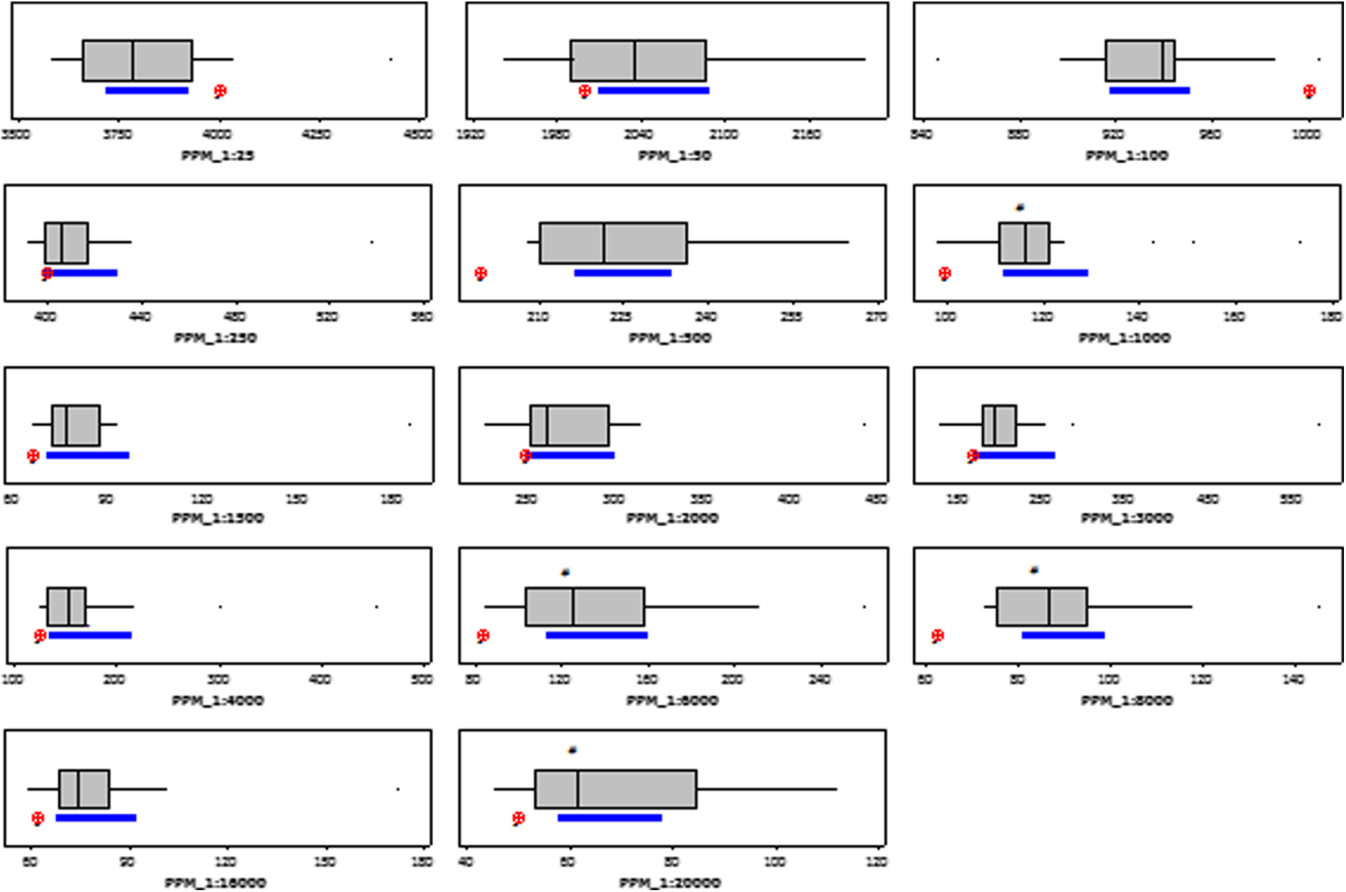
**Test dilutions t-test boxplots.** Expected PPM (red dot), 95% confidence interval from PPM results (blue line). The asterisk (*) indicates outliers while hash (#) indicates dilutions producing a significant different PPM than expected.

**Figure 3:**
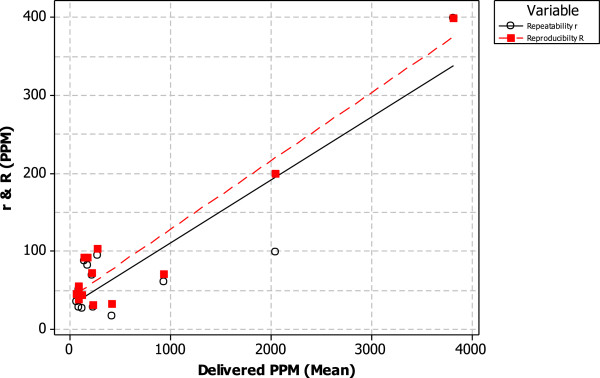
Scatterplot of repeatability (r) (white circle) and reproducibility (R) (red square).

An initial range of 14 dilutions from 1:25 to 1:20,000 were selected to evaluate the actual dilution delivery in PPM units using reference methane gases (Table 2). The data in Figure 2 graphically represents the results from the statistical 1-sample t-test analysis performed comparing PPM obtained per dilution (box plot) against the expected PPM (red dot), the analysis did not incorporate the ±10% tolerance accepted for machinery measurement variation and was, therefore, added to the expected PPM value afterwards [31]. Results indicate that in the majority of the cases (10 out of 14 dilutions) the dilution delivery was as expected when the ±10% tolerance was included in the analysis. There were four dilutions where the statistical analysis (1-sample t-test) showed a statistically significant difference between measured and expected PPM (including ±10% measurement variation), those dilutions are identified in Figure 2 with a hash (#) (1:1,000, 1:6,000, 1:8,000 and 1:20,000) and were not taken into consideration for the assessment of WMCS in the *in vitro* γH2AX assay.Figure 3 represents the repeatability and reproducibility results indicating the precision of the smoking machine dilution performance within the same experiment and in different experiments respectively. The repeatability and reproducibility increased linearly with concentration as expected.

### WMCS genotoxicity assessment

**Figure 4:**
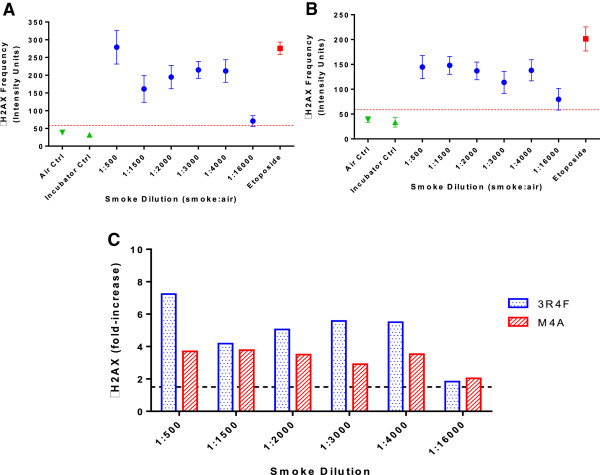
**γH2AX frequency mean ± SD after 3 h exposure to WMCS from reference cigarette. [A]** 3R4F, **[B]** M4A. Circle (-●-) represents WMCS results, square (-■-) represents positive control etoposide (1 mM final), triangles (-▼- and -▲-) represents negative controls, air and incubator controls respectively and dotted red line represents the 1.5-fold increase over the air control indicating the threshold of genotoxic response. **[C]** γH2AX fold-induction for both reference cigarettes 3R4F (blue) and M4A (red), dotted line indicates genotoxic level (>1.5-fold γH2AX response).

Initial range finder experiments showed that 3 hour exposures to WMCS from 3R4F cigarettes at dilutions more concentrated than 1:500 produced tar depositions, this effect was considered equivalent to precipitation. Only dilutions greater than 1:500 were included in further experiments. Both reference cigarettes 3R4F and M4A produced a significant increase in γH2AX frequency (above 1.5-fold increase) compared to the air-treated control in all the dilutions tested (Figure 4). In all experiments the positive control etoposide produced an increase greater than 1.5-fold compared to the air-treated control (Figure 4A and B). Relative Cell Counts (RCC) for all results presented were above the acceptance limit of toxicity (RCC > 25%) and therefore no cytotoxic-driven genotoxicity was observed (Table 3).Figure 4A illustrates the response produced after 3 hour exposure to 3R4F WMCS. A variation in the response can be observed between the most concentrated WMCS (1:500) and the most diluted WMCS dilution (1:16,000). However, the linear regression model indicates a low correlation between the dose and the response (Pearson coefficient, r = -0.53). Figure 4B showed the results obtained after 3 hour exposure to M4A WMCS. In this case, a variation in the response can only be observed at the most diluted WMCS dilution tested (1:16,000). The linear regression model produced a low correlation between the dose and the response (Pearson coefficient r = -0.44). Figure 4C graphically represents the fold-induction results from both reference cigarettes. In general, 3R4F WMCS exposure seems to have a more potent genotoxic effect compared to M4A WMCS exposure, especially at the most concentrated dilution 1:500.

## Discussion

The main objective of this study was to optimise the novel *in vitro* γH2AX by HCS for the genotoxicity assessment of aerosols. During the optimisation, the genotoxic potential in the form of γH2AX induction from various dilutions of WMCS of two reference cigarettes were tested and differences in the response evaluated.

The cell system selected was the BEAS-2B cell line, a human-derived cell line from the lung, the first target tissue of inhaled aerosols. The non-tumorigenic human-derived BEAS-2B cell line was isolated from normal human epithelium and immortalised by virus infection [22]. The normal phenotype and wild-type p53 status support the use of this cell line in DNA damage studies [33-35]. BEAS-2B cells, however, lack normal metabolic activity for the majority of cytochrome P450 family, an essential factor for the phase I bioactivation of some cigarette smoke toxicants such as 4-(methylnitrosamino)-1-(3-pyridyl)-1-butanone (NNK) [36]. The limitation in the metabolic capability of the cell line would need to be considered in future experimental designs i.e. including an external source of metabolic activation in part of the experiments to have a more comprehensive genotoxicity evaluation of the WMCS.

The Borgwaldt RM20S® smoking machine has been extensively used for the *in vitro* evaluation of WMCS [5,24,37]. Although, some QC analyses have been reported for the accurate performance of the syringes [25,26] further QC tests for the accurate performance of the programmed dilutions have proved necessary. Our results in this study indicate that not all of the programmed dilutions deliver the expected amount of reference gas in PPM (Figure 2). We have observed that more diluted dilutions seem to produce less accurate deliveries; this effect could be caused by the smoking machine dilution programming. The smoking machine performs a multistep process to dilute WMCS with laboratory conditioned air, the process requires the programming of more dilution steps for more diluted dilutions, hence, the potential for more variation. The discrepancy between expected and delivered aerosol could affect the exposure to the cell cultures and ultimately the outcome of the assay. The same approach could be applied in the future to the particulate phase expected in the different dilutions employing Quartz Crystal Microbalances (QCM) previously described for this aerosol exposure system [38]. Nevertheless, the smoking machine performance has shown an overall good reproducibility and repeatability from dilutions delivering 50 PPM or above as can be seen in Figure 3. The performance of syringes and dilutions can be carried out using the same methodology and apparatus already in place for the standard QC checks. Moreover, the extended QC check could easily be incorporated into the routine service of the Borgwaldt RM20S® smoking machine.

The γH2AX results obtained during the assessment of two reference cigarettes seem to indicate that the *in vitro* γH2AX assay by HCS was able to detect the genotoxic potential of WMCS, however the correlation between the dose and the response was low for both reference cigarettes evaluated in this study across all the tested dilutions (Figure 4). Nevertheless, the γH2AX response obtained after BEAS-2B cells were exposed to a range of 3R4F WMCS dilutions for 3 hours was in general more potent than the response obtained for M4A WMCS and can be visually observed in Figure 4C. If the genotoxicity response was primarily associated to the gas phase we would have expected a better γH2AX dose–response correlation with the different dilutions tested. Therefore, we have considered that the particulate phase may have a significant effect in driving the genotoxic potential. This could be further investigated by characterising the particulates deposited at different dilution levels with tools such as the QCM balance mentioned earlier in this discussion.

It is important to notice that 3 hour continuous exposure as recommended by ICH guidelines [30] could be the longest exposure time a submerged monolayer culture might be exposed at the ALI. In our experiments, the cell cultures were immediately fixed after the exposure to evaluate the DNA damage in the form of γH2AX. Pilot experiments were conducted where the cell cultures were left to recover for a further 24 hours submerged in media to evaluate potential DNA repair after the acute 3 hour exposure. The proliferation of the BEAS-2B cells was greatly affected in WMCS and air control samples. Interestingly, the same effect was not observed in incubator control cultures where the humidity is maintained at a higher level (data not shown). We concluded that for *in vitro* assays using submerged cultures as cell systems, 3 hour exposure at the current conditions of ALI exposure system would cause irreversible damage due to drying as opposed to aerosol exposure.

Following the optimisation described in this study, further investigations employing different exposure times, a larger number of products and an external source of metabolic activation would be necessary to support the applicability of the *in vitro* γH2AX assay for the evaluation of tobacco products in aerosol exposure. Future work could also carry out an in-depth characterisation on the effect that product variations such as different tobacco blends have in γH2AX induction to understand the differences in response.

Nevertheless, the optimisation performed here could also be applied to the genotoxicity evaluation of other aerosols such as aerosolised drugs, pollutants and cigarette smoke toxicants present in the gas phase (e.g. benzene).

## Conclusions

Overall, the *in vitro* γH2AX assay by HCS could be used to evaluate WMCS in cell cultures at the ALI. Additionally, the extended characterisation of the exposure system indicates that assessing the performance of the dilutions could improve the existing routine QC checks.

## Abbreviations

ALI: Air liquid interfase; BEGM: Bronchial epithelial growth medium; CSC: Cigarette smoke condensate; CSE: Cigarette smoke extract; DSB: Double strand break; HCS: High content screening; ISO: International standard organization; PM: Particulate matter; PPM: Parts per million; QCM: Quartz crystal microbalance; RCC: Relative cell count; RH: Relative humidity; SD: Standard deviation; WHO FCTC: World health organisation framework convention on tobacco control; WMCS: Whole mainstream cigarette smoke.

## Competing interests

The authors declare that they have no competing interests.

## Authors’ contribution

CGC, GE and CM designed the study. CGC conducted all the experiments. Data analysis was performed by CGC and GE. CGC wrote the manuscript with GE and CM support. CM and AA provided scientific support. All authors approved the final manuscript.

## Pre-publication history

The pre-publication history for this paper can be accessed here:

http://www.biomedcentral.com/2050-6511/15/41/prepub
